# Emollient ditrimethylolpropane tetracaprylate penetrates the stratum corneum and maintains hydration without disrupting lipid structure

**DOI:** 10.1038/s41598-026-52849-1

**Published:** 2026-06-05

**Authors:** Yukiko Suzuki Uemura, Naoko Hanada Yamazaki, Manaka Honda, Toshinori Saida, Keiichi Oyama, Yoshihiro Tokudome

**Affiliations:** 1 Laboratory of Cosmetic Sciences, Graduate School of Science and Engineering, Saga University, 1 Honjo, Saga, 840-8502 Japan; 2 Department of Fine Chemicals, The Nisshin OilliO Group, Ltd., 1 Shinmori-cho, Isogo-ku, Yokohama, 235-8558 Japan

**Keywords:** Skin hydration, Oil-based moisturizer, Occlusion agent, Skin permeability, Stratum corneum lipids, Health care, Materials science

## Abstract

Emollients are essential for skin hydration, yet the relationship between their penetration into the stratum corneum (SC) and moisturizing efficacy remains unclear. This study aimed to elucidate the mechanism of the ester oil, ditrimethylolpropane tetracaprylate (DTTC), focusing on its persistent hydration effect and its interaction with SC lipids. We compared DTTC with other emollients and an occlusive benchmark using in vivo assays, permeability studies, and differential scanning calorimetry (DSC). In vivo, DTTC produced a superior and sustained hydrating effect after wash-off, without disrupting barrier function. Permeability analyses confirmed that DTTC penetrates and is retained within SC, in contrast to less persistent or non-penetrating agents. Furthermore, DSC showed that DTTC integrates into a model SC lipid matrix with high compatibility and minimal structural disruption. We conclude that the superior performance of DTTC reflects a dual mechanism: effective penetration and retention within SC, together with structurally compatible integration into the intercellular lipid matrix. This highlights a pathway to long-lasting hydration beyond simple surface occlusion and provides a basis for the rational design of advanced emollients.

**Supplementary Information:**

The online version contains supplementary material available at 10.1038/s41598-026-52849-1.

## Introduction

Dry skin is a widespread cosmetic concern, with up to one-third of the global population reporting this condition^1^. To manage this condition, skincare science uses moisturizing agents that are typically classified into three functional categories: humectants, occlusives, and emollients. Humectants, such as glycerin, attract and retain water within the stratum corneum (SC), while occlusives, such as petrolatum, form a hydrophobic film on the skin surface to physically reduce transepidermal water loss (TEWL)^2^. In contrast, emollients—the focus of this research—are lipidic substances that primarily fill intercellular voids within the SC lipid matrix, contributing to skin softness, flexibility, and local hydration. Although emollients can also reduce TEWL, their mechanisms extend beyond simple film formation^2^.

The emollient category encompasses a wide range of chemical structures, including natural triglycerides (fats and oils), waxes, fatty acids, and synthetic esters. This study focuses specifically on synthetic ester emollients, which are produced by reacting an alcohol with a carboxylic acid and are distinct from naturally occurring triglycerides or wax esters. These synthetic esters are valued for their customizable sensory profiles and stability.

Although some emollients can penetrate SC^2,3^, the relationship between their penetration behavior and moisturizing effects remains poorly understood. Traditional discussions on lipid-based skincare ingredients have focused primarily on surface effects, such as the barrier-forming properties of occlusives or the surface-smoothing action of emollients^4–9^. Moreover, research on compounds such as hydrocarbon oils and certain plant-derived triglycerides suggest that these materials may be largely confined to the uppermost SC layers, limiting their direct interaction with deeper skin layers^4–9^. This raises an intriguing question about which types of emollients, including synthetic esters, achieve deeper penetration, and whether this contributes significantly to skin hydration.

To address this question, it is essential to determine where these compounds reside within the skin. Advanced analytical techniques, such as imaging mass spectrometry, enable spatial mapping of topically applied substances^10,11^. Although few studies have examined synthetic esters using these approaches, applying such methodologies could provide critical insights into moisturizing mechanisms by directly linking penetration depth with biological effects.

A crucial consideration for any substance that penetrates SC is its interaction with intercellular lipids. These lipids organize into highly ordered lamellar structures, and their packing phase is a key determinant of the skin’s barrier properties^12,13^. The tightly packed orthorhombic phase is associated with low water permeability and a robust barrier, whereas the less ordered hexagonal phase corresponds to increased permeability and impaired barrier function^14,15^. Consequently, exogenous substances that interact with these lipids can influence barrier integrity; for example, inducing a phase transition from orthorhombic to hexagonal packing would compromise the barrier. A key question therefore arises: can an emollient penetrate SC to provide a lasting hydration benefit without adversely affecting this delicate lipid architecture?

To address these questions, this study first conducted a comparative evaluation of various lipid-based agents, including several synthetic esters and a hydrocarbon oil (as an occlusive benchmark), to investigate their moisturizing effects and permeability. In this initial screening, ditrimethylolpropane tetracaprylate (DTTC), a synthetic polyol ester, emerged as a particularly promising candidate owing to its strong and sustained hydration effects. We therefore focused subsequent investigations on elucidating the mechanisms underlying DTTC performance. Specifically, we aimed to: (1) confirm its sustained hydration effect after removal, thereby distinguishing its action from a purely occlusive effect; (2) quantify its penetration profile within SC and visualize its distribution; and (3) assess its influence on the thermal behavior of model SC lipid systems as an indicator of lipid interactions. Our findings establish DTTC as a high-performing emollient and provide foundational evidence linking the penetration of a specific synthetic ester to its hydrating efficacy. This work offers insights into its interactions with SC lipids and provides a basis for further mechanistic studies to inform advanced emollient design.

## Results

### In vivo skin hydration assay

**Fig. 1:**
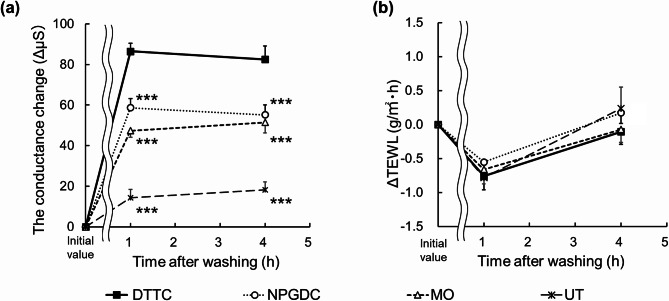
Effects of lipid-based agents on the SC moisture content and transepidermal water loss (TEWL). Black squares, white circles, white triangles, and crosses indicate DTTC, neopentylglycol dicaprate (NPGDC), mineral oil (MO), and untreated area (UT), respectively. (**a**) Change in conductance measured using a SKICON-200EX device after topical application of the tested oil samples and subsequent wash-off. (**b**) TEWL after topical application of the tested oil samples and subsequent wash-off. Values are expressed as mean ± SE. Statistical significance was assessed using one-way ANOVA followed by Dunnett’s multiple comparisons test, with DTTC as the control group: *p < 0.05, **p < 0.01, ***p < 0.001; *n* = 23.

The practical application test was performed using oil samples selected based on preliminary investigations. The tested oils generally increased SC moisture content even after their removal from the skin surface. DTTC produced a significantly greater increase in SC moisture content compared with the other samples and the untreated control (Fig. 1a). In contrast, TEWL values were generally comparable among the tested oils (Fig. 1b).

### Quantitative analysis of skin permeability (GC-FID)

**Fig. 2:**
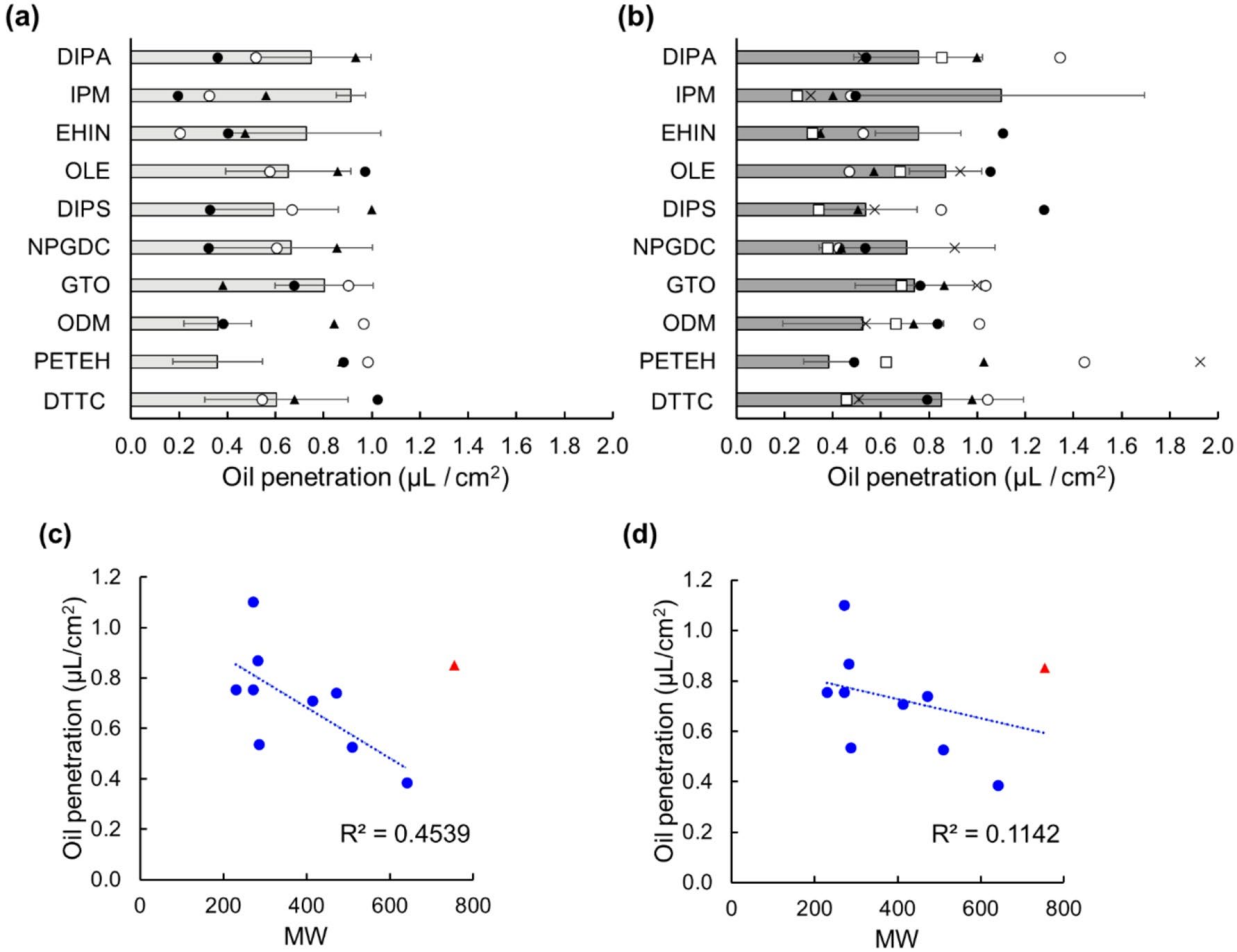
Ex vivo skin penetration profiles and molecular-weight dependence of lipid-based agents. (**a**,**b**) Time-dependent penetration of oils (DIPA, IPM, EHIN, OLE, NPGDC, ODM, PETEH, DTTC, DIPS, and GTO) at 12 h (*n* = 3) and 24 h (*n* = 5), respectively. Data points are indicated by an upward-pointing triangle (*n* = 1), open circle (*n* = 2), filled circle (*n* = 3), open square (*n* = 4), and cross (*n* = 5). (**c**,**d**) Relationship between molecular weight and skin penetration at 24 h. Conventional lipid-based agents are shown as blue circles and DTTC as a red triangle. Regression lines and corresponding R^2^ values are shown for conventional lipid-based agents alone (**c**) and for all lipid-based agents (**d**), respectively.

For diisopropyl adipate (DIPA), isopropyl myristate (IPM), ethylhexyl isononanoate (EHIN), oleic acid (OLE), NPGDC, octyldodecyl myristate (ODM), pentaerythrityl tetraethylhexanoate (PETEH), and DTTC, penetration amounts were higher at 24 h than at 12 h. By contrast, diisopropyl sebacate (DIPS) and glyceryl trioctanoate (GTO) exhibited higher penetration at 12 h than at 24 h (Fig. 2a, b). For all samples, the cumulative amount permeated at 24 h was less than twice the value measured at 12 h. Analysis of molecular weight versus skin penetration at 24 h revealed an inverse relationship for conventional oils (R^2^ = 0.4539), where a higher molecular weight was associated with lower skin penetration. However, when DTTC was included in the analysis, the coefficient of determination decreased to R^2^ = 0.1142, indicating that DTTC penetrated more efficiently than conventional lipid-based agents of comparable molecular weights (Fig. 2c, d).

### Skin permeability visualization with MS imaging analysis

**Fig. 3:**
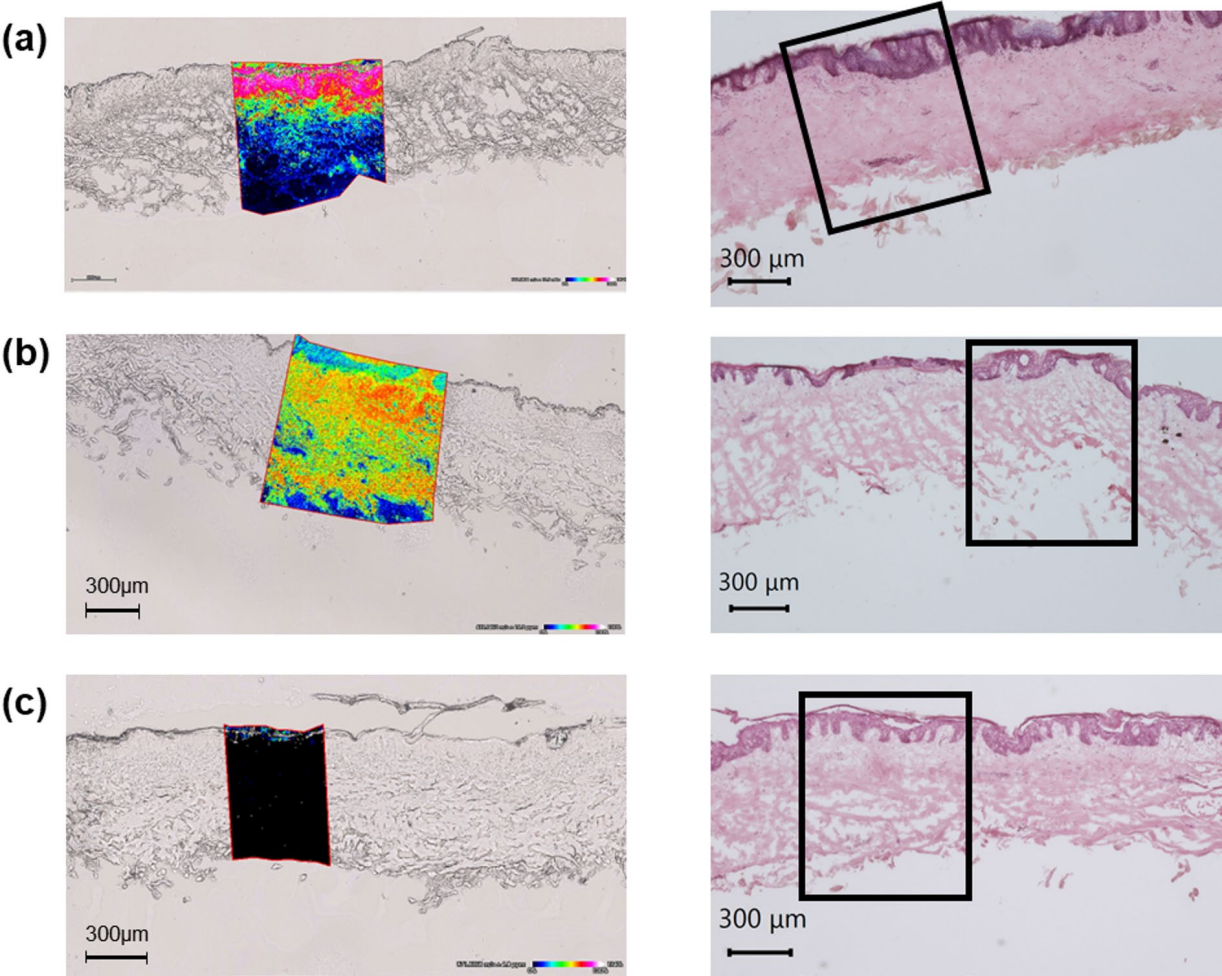
Mass imaging spectrometry (MSI) of each oil sample. Optical images of a tissue section from skin treated with (**a**) DTTC, (**b**) NPGDC, or (**c**) TMPIS.

As a preliminary in vivo assessment prior to the ex vivo MSI experiments, tape-stripped SC was analyzed by Nano-PALDI MSI. After washing, characteristic ions attributable to glyceryl oleate (GO), DTTC, NPGDC, triolein (TO), trimethylolpropane triisostearate (TMPIS), and squalene (SQ) were detected on the stripped SC sheets and declined over time (Supplementary Fig. S1), whereas no corresponding signals were observed at untreated sites. These results confirmed surface deposition and time-dependent clearance, supporting subsequent ex vivo permeation/MSI experiments. Based on chemical structures, the *m/z* of each sample was calculated, and the expected *m/z* values were observed. TMPIS (*m/z* 971.84, sodium adduct) localized only on the SC surface, indicating limited penetration (Fig. 3a). Conversely, DTTC (*m/z* 777.59, sodium adduct) and NPGDC (*m/z* 435.35, sodium adduct) penetrated into deeper skin layers (Fig. 3b, c). Notably, comparison of DTTC and NPGDC revealed that DTTC tended to be retained in the upper skin layers, whereas the smaller molecule NPGDC appeared to penetrate more deeply and with broader distribution.

### Thermal behavior of lipid matrix models

**Fig. 4:**
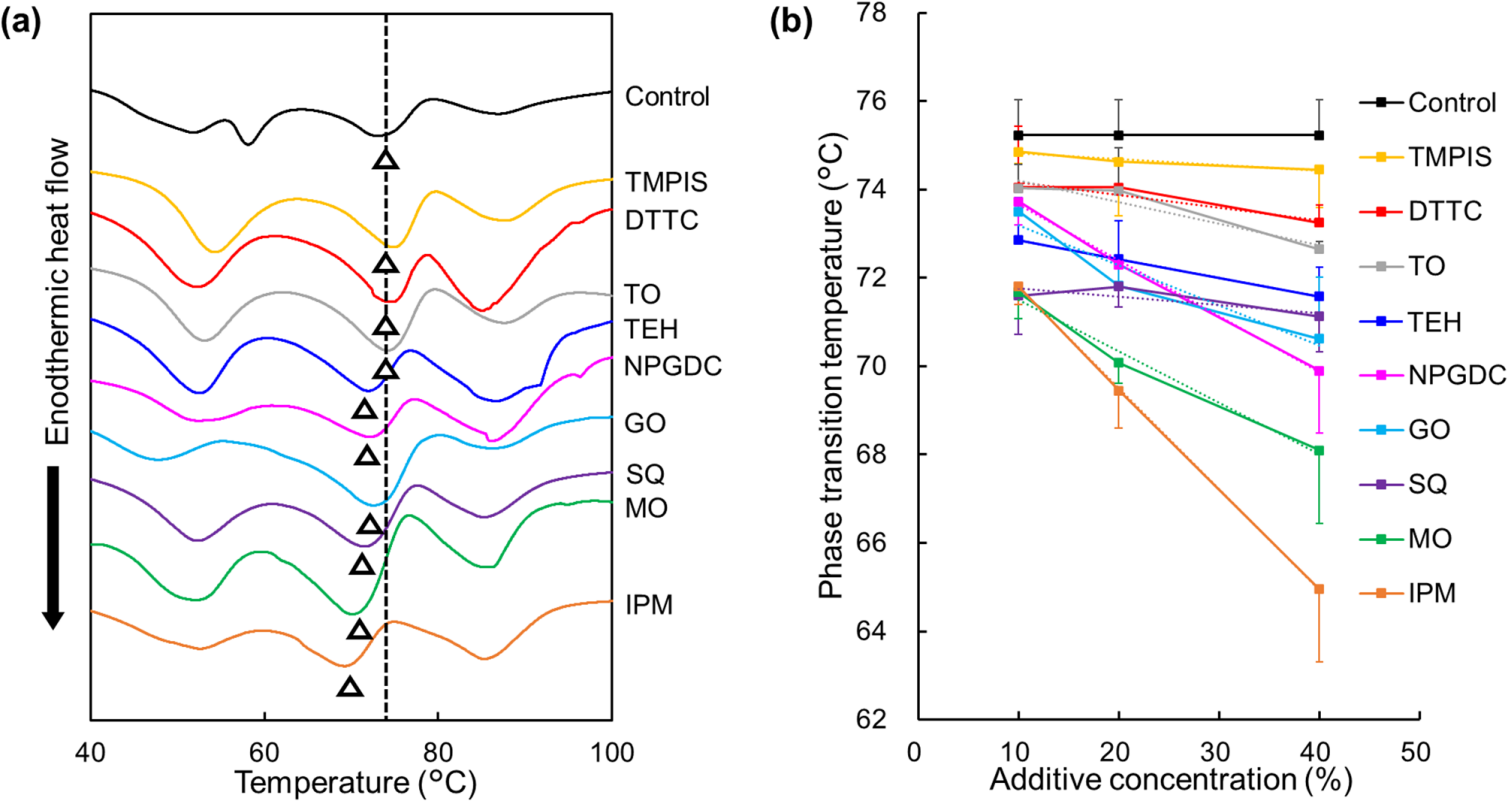
Effects of oil samples on the SC lipid phase transition peak and its concentration dependence. (**a**) Thermal behavior of the SC intercellular lipid matrix. Triangles indicate the phase transition peak after addition of the oil sample (20%). (**b**) Concentration dependence of the phase transition peak shift for each tested oil sample. Data are expressed as mean ± SD; *n* = 4.

The SC intercellular lipid phase transition peak, typically observed at 70–80 °C^16^, shifted to lower temperatures after addition of TEH, NPGDC, GO, SQ, MO, and IPM. By contrast, the addition of TMPIS, DTTC, and TO caused minimal shifts (Fig. 4a). To examine concentration effects, systems containing 10, 20, and 40% oil were analyzed (Table 1).

**Table 1:** Phase transition temperature of SC intercellular lipids upon the addition of 10, 20, and 40% oil sample.

Sample	Phase transition temperature (℃)		
	10%	20%	40%
Control	75.2 ± 0.81	75.2 ± 0.81	75.2 ± 0.81
TMPIS	74.9 ± 0.26	74.6 ± 1.2	74.5 ± 0.86
DTTC	74.1 ± 1.4	74.1 ± 0.55	73.3 ± 0.40
TO	74.9 ± 0.55	74.0 ± 1.0	72.7 ± 0.17
TEH	72.9 ± 0.60	72.4 ± 0.87	71.6 ± 0.67
NPGDC	73.7 ± 0.54	72.3 ± 0.41	69.9་1.4
GO	73.5 ± 0.31	71.8 ± 0.51	70.6 ± 1.4
SQ	71.6 ± 0.88	71.8 ± 0.45	71.1 ± 0.80
MO	71.7 ± 0.59	70.1 ± 0.47	68.1 ± 1.7
IPM	71.8 ± 0.41	69.5 ± 0.86	65.0 ± 1.7

The concentration dependence of the phase transition differed across the tested oil samples. The absolute slopes of the linear fits for each oil sample were as follows: IPM (|slope| = 0.23, molecular weight (MW) = 270), NPGDC (|slope| = 0.13, MW = 413), MO (|slope| = 0.12, MW cannot be calculated), GO (|slope| = 0.091, MW = 356), TO (|slope| = 0.049, MW = 885), TEH (|slope| = 0.043, MW = 471), DTTC (|slope| = 0.029, MW = 755), SQ (|slope| = 0.018, MW = 410), and TMPIS (|slope| = 0.0013, MW = 933). Among these, IPM, NPGDC, and MO exhibited stronger concentration dependence, whereas DTTC, SQ, and TMPIS showed weaker dependence. Overall, synthetic ester oils with lower MW tended to exhibit stronger concentration dependence (Fig. 4b).

**Fig. 5:**
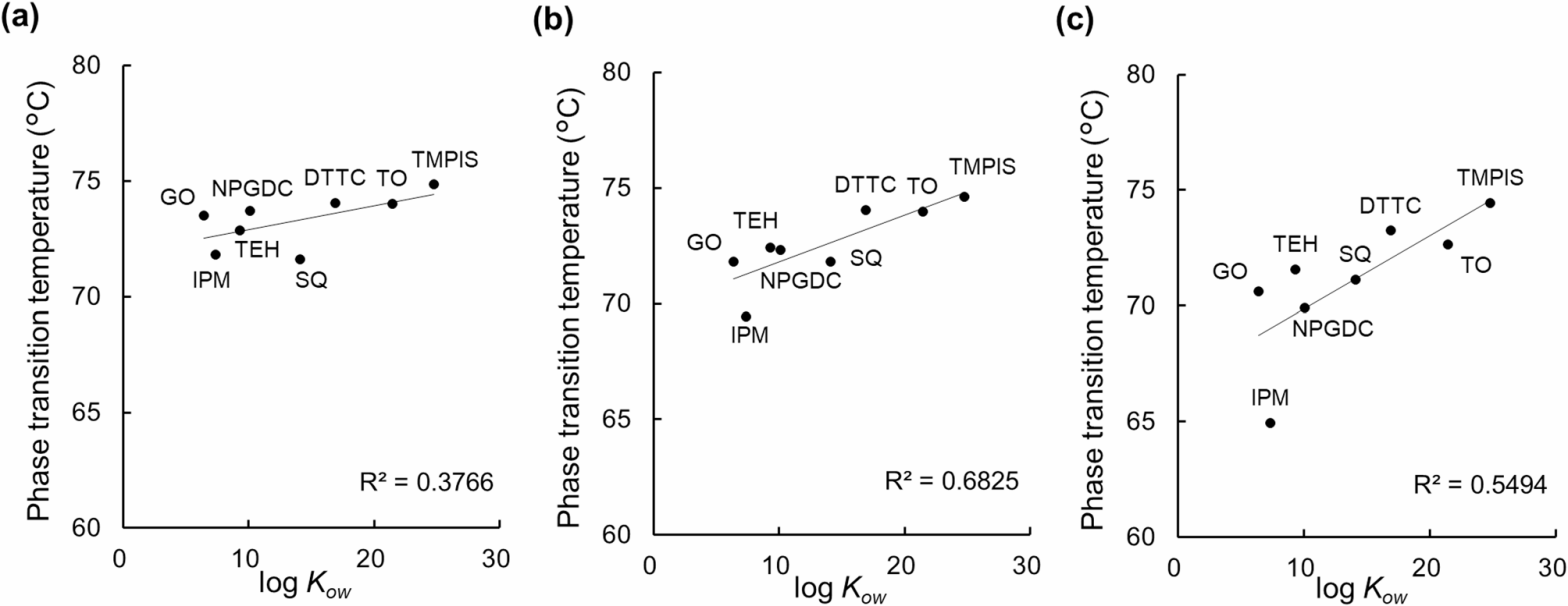
Correlation between the phase transition temperature of SC intercellular lipids and log *K*_*ow*_ of oil samples. Systems containing (**a**) 10%, (**b**) 20%, and (**c**) 40% oil samples.

Across systems containing 10, 20, and 40% oil, the phase transition temperature correlated with the log *K*_*ow*_ values of the oils (Fig. 5a–c), with coefficients of determination R^2^ = 0.38, 0.68, and 0.55, respectively. Additional correlations were also observed between the phase transition temperature and MW, relative permittivity, and the inorganic/organic balance (IOB) values of the oil samples (Supplementary Fig. S2–S4).

## Discussion

Current understanding of lipid-based moisturizing agents often emphasizes surface effects, including barrier-forming occlusion by hydrocarbons and the surface-smoothing action of emollients^4–9^. This study aimed to examine mechanisms beyond the surface by investigating whether emollient penetration contributes to sustained skin hydration. We therefore performed a comparative evaluation of several ester oils relative to a hydrocarbon oil, which served as a classic occlusive benchmark.

The in vivo skin hydration assay revealed that the application of lipid-based agents generally increased the SC moisture content, and this effect persisted even after washing the skin (Fig. 1a). Skin surface hydration measurements (e.g., corneometry) can be influenced by residual substances or changes in the dielectric properties of the SC surface. However, by applying a standardized wash-off protocol to all sites, including the untreated control, we sought to minimize contributions from surface residues and to better isolate the effects arising from interactions within SC. Among the tested oils, DTTC produced a significantly greater increase in skin hydration compared with the other samples and the untreated control. This sustained effect is consistent with a mechanism distinct from transient surface occlusion. TEWL measurements offer further context for these findings (Fig. 1b): for all tested oils, TEWL values remained close to baseline throughout the experiment, indicating that application and subsequent wash-off did not measurably impair barrier function. We therefore led us to hypothesize that DTTC’s exceptional performance is linked to its ability to penetrate and remain within SC.

To test this hypothesis, we quantified the skin permeability of the compounds. It is important to acknowledge that the penetration of emollients into the skin, particularly into deeper skin regions, remains a subject of considerable debate. Many studies, especially those employing robust non-invasive techniques such as confocal Raman microscopy (CRM), have shown that the most common lipid-based agents, including vegetable oils, paraffin oil, and petrolatum, are largely confined to the superficial layers of the SC, typically penetrating less than ~ 11 μm^17,18^. Similarly, even smaller non-polar molecules, such as those found in essential oils, exhibit very limited penetration into the viable epidermis^19^. Some previous reports suggesting deeper penetration may have been influenced by analytical artifacts^17,18^.

While numerous studies have established that certain emollients can act as penetration enhancers to facilitate the delivery of other active ingredients into the skin^2,20^, there remains limited systematic research focusing on the penetration of the emollient molecules themselves and how this intrinsic behavior contributes to skin benefits such as hydration. Most existing studies are also limited to a small and specific set of traditional oils^17,18^. Nevertheless, penetration and volume occupation within the SC are functionally significant. For example, recent work by Berkey et al. demonstrated that such volume occupation contributes to a reduction in biomechanical skin stress^21^, suggesting a plausible secondary benefit of DTTC penetration in addition to enhanced hydration. Our GC-FID analysis showed that DTTC penetrated the skin more effectively than other tested lipid-based agents of similar MW (Fig. 2c, d). Although skin penetration is generally more efficient for compounds with MW < 500^22^, DTTC deviated from this trend. This deviation suggests that specific structural and physicochemical properties facilitate DTTC transport. Structurally, DTTC is a polyol fully esterified with medium-chain fatty acids. Its di-trimethylolpropane-derived core accommodates four polar ester linkages within a compact molecular framework, yielding a notably high ratio of ester groups to hydrocarbon chain length relative to simpler lipids such as triglycerides. Consequently, DTTC exhibits higher polarity—as reflected by its relative permittivity of 4.94—than would be expected for a molecule of its size and lipidic character. We hypothesize that this feature enables DTTC to partition efficiently between lipid and aqueous microdomains of SC. This interpretation is consistent with established principles in transdermal drug delivery, where the ability of penetration enhancers to partition into both lipid and polar domains is considered crucial for their efficacy^23–25^.

However, it is important to note that the GC-FID method quantifies the total amount of compound penetrating the full-thickness skin sample and does not distinguish between the SC and the deeper regions. To investigate spatial distribution, MSI was employed, which further supported these findings by showing DTTC in deeper SC layers, whereas TMPIS remained largely on the surface (Fig. 3a, b). While MSI provides high sensitivity and spatial information, it has limitations in absolute quantification and is constrained by the use of excised skin. To further validate these findings in a more dynamic system, future studies should employ non-invasive in vivo imaging techniques. Methods such as laser scanning microscopy and RCM would enable real-time visualization of emollient distribution within skin layers and allow assessment of their effects on SC morphology, including reductions in micro-scales and surface irregularity, as reported for other moisturizers^26,27^.

However, penetration alone is not sufficient; interaction with the intercellular lipid matrix is also critical. DSC analysis provides insight into this interaction. We recognize that shifts in lipid phase-transition temperatures can arise from multiple factors, including not only substance penetration but also surface plasticization of the lipid phase or changes in its hydration state. Nevertheless, DTTC integrated into the model lipid matrix with minimal disruption of its thermal behavior (Fig. 4a), indicating a high degree of structural compatibility. Unlike smaller esters such as IPM and NPGDC, which induced more pronounced changes at lower concentrations, DTTC exhibited high compatibility with the lipid organization (Fig. 4b). This interpretation is supported by the imaging results. As noted in the Results section, MSI revealed that although both DTTC and NPGDC penetrated SC, their distribution patterns differed significantly. The smaller molecule NPGDC penetrated more deeply and diffusely. In contrast, DTTC was largely retained within SC, with some presence detected in deeper skin regions (Fig. 3b, c). This pattern suggests that DTTC is not merely traversing SC but is actively integrating within its structure. These observations support a plausible mechanism for DTTC’s superior performance. We propose that, whereas smaller molecules such as NPGDC may diffuse more deeply and broadly, DTTC’s molecular architecture and size favor residence within the intercellular spaces of the lipid matrix.

This “integrative” behavior suggests that DTTC compatibly occupies free volume within the lipid organization, thereby increasing SC water-retaining capacity (consistent with the sustained hydration in Fig. 1a) without compromising structural integrity or inducing a marked change in TEWL. This proposed mechanism is consistent with the imaging data (Fig. 3) and with the observation that no single physicochemical descriptor fully predicts this behavior (Fig. 5, Supplementary Fig. S5). Collectively, these results support the view that DTTC’s dual function—effective penetration coupled with structural compatibility—is governed by a specific combination of molecular architecture and balanced polarity, rather than by lipophilicity alone.

In summary, our findings support a dual mechanism underlying DTTC’s superior performance: it provides a lasting moisturizing effect not by forming a simple surface film, but by (1) effectively penetrating SC and (2) compatibly integrating with the intercellular lipid matrix, thereby enhancing its water-retention capacity. This mechanism distinguishes DTTC from classic occlusives and other less effective emollients.

Future studies should further elucidate these molecular-level mechanisms. Small- and wide-angle X-ray scattering (SAXS/WAXS) and attenuated total reflectance Fourier-transform infrared spectroscopy (ATR-FTIR) would be valuable for defining effects on lamellar organization and lateral packing of the SC lipids. In addition, molecular dynamics simulations will probe the energetic and conformational determinants of DTTC integration into the lipid matrix. Together, these approaches should provide a more definitive link between an emollient’s molecular structure and its hydrating efficacy, guiding the rational design of next-generation skincare ingredients.

## Methods

### Materials

DTTC (CAS: 18367-21-0) was synthesized via esterification of ditrimethylolpropane with caprylic acid. Briefly, caprylic acid and ditrimethylolpropane were heated to > 200 °C under a nitrogen atmosphere in the presence of a metal catalyst, and esterification proceeded with continuous removal of generated water. After completion, the product was purified using conventional methods to obtain the target ester. The test chemicals used in the assessments were cosmetic-grade ingredients (Table 2). Ceramide III (95.9%) was purchased from Evonik Degussa (Essen, Germany). Palmitic acid was obtained from MERCK (Darmstadt, Germany). Cholesterol was supplied by Nacalai Tesque (Kyoto, Japan). All other reagents were of analytical grade and used without further purification.

**Table 2:** Cosmetic ingredients used for assessments.

	INCI	MW	Relative Permittivity	Supplier
DIPA	Diisopropyl adipate	230	-	FUJIFILM wako pure chemical corporation
DIPS	Diisopropyl sebacate	286	4.67	Industrial Química Lasem S.A.U.
DTTC	Ditrimethylolpropane tetracaprylate	755	4.94	The Nisshin OilliO Group, Ltd.
EHIN	Ethylhexyl isononanoate	271	3.40	Industrial Química Lasem S.A.U.
GTO	Glyceryl trioctanoate	471	3.87	The Nisshin OilliO Group, Ltd.
GO	Glyceryl oleate	356	-	Kanto Chemical Co., Inc.
IPM	Isopropyl myristate	271	3.28	FUJIFILM Wako Pure Chemical Corporation
MO	Mineral oil	-	2.13	Sonneborn LLC
NPGDC	Neopentylglycol dicaprate	413	3.40	The Nisshin OilliO Group, Ltd.
ODM	Octyldodecyl myristate	509	3.26	Nikko Chemicals Co., Ltd.
OLE	Oleic acid	282	2.36	Merck KGaA
PETEH	Pentaerythrityl tetraethylhexanoate	641	2.99	The Nisshin OilliO Group, Ltd.
SQ	Squalene	410	-	Tokyo Chemical Industry Co., Ltd.
TEH	Triethylhexanoin	471	4.19	The Nisshin OilliO Group, Ltd.
TMPIS	Trimethylolpropane triisostearate	933	2.93	The Nisshin OilliO Group, Ltd.
TO	Triolein	885	-	Tokyo Chemical Industry Co., Ltd.

### In vivo skin hydration study

A total of 23 healthy subjects (16 females, 7 males; 20–60 years) participated after providing written informed consent. The study protocol was approved by the Osaka Shoin Women’s University Research Ethics Committee (Application No. 23–26). All methods were performed in accordance with relevant guidelines and regulations, including the Declaration of Helsinki. Measurements were conducted under controlled conditions (indoor relative humidity 50 ± 10% and temperature 23 ± 2 °C). At the start of the experiments, subjects’ forearms were gently cleansed with soap and allowed to equilibrate for 30 min. Skin measurement sites were marked as 2.5 × 2.5 cm² areas on the inner forearms of each subject. After measuring the initial value, 28 µL of each sample was applied for 1 h, with identical treatments across subjects. All measurement sites, including a designated untreated (UT) area, were then washed with moisturizer-free soap containing potassium laurate, potassium myristate, potassium palmitate, and potassium stearate. Measurements were repeated at 1 and 4 h after washing. The SC moisture content was measured using a SKICON-200EX device (Yayoi, Tokyo, Japan). DTTC, NPGDC, and MO were evaluated, with UT serving as a negative control. Because the number of application sites per subject was limited, these compounds were selected from commonly used base oils with similar viscosities to represent distinct expected penetration profiles: MO as a low-penetrating hydrocarbon oil benchmark and NPGDC as a lower-molecular-weight ester with higher expected penetration.

### Ex vivo skin permeation studies

Permeation tests were performed using human abdominal skin (0.4 mm thickness; Biopredic Int., Saint-Grégoire, France) under permit AC-2013-1754 granted by the French Ministry of Higher Education and Research to Biopredic Int. (authorizing the acquisition, processing, sale, and export of human biological material for research purposes). An 8 mm inner-diameter glass tube was affixed to the surface of the excised human skin, and 100 µL of each sample was applied within the tube. Samples were incubated at 32 °C for the designated durations, depending on the experimental conditions. After incubation, excess oil was carefully removed, and the treated skin samples were subjected to further analyses.

### Quantitative analysis of skin permeability (GC-FID)

The amount of oil that penetrated the skin was quantified via gas chromatography with a flame ionization detector (GC-2014, Shimadzu, Kyoto, Japan). For this analysis, the following oils were evaluated: DIPA, IPM, EHIN, OLE, DIPS, NPGDH, GTO, ODM, PETHE, and DTTC.

### Sample preparation for MS imaging (MSI)

Spatial distribution of each sample within the skin was visualized through imaging mass spectrometry (MSI). Following initial incubation, the skin samples were further incubated at 32 °C for 1 h. The treated skin was embedded in a super cryo-embedding medium (SCEM, SECTION-Lab, Hiroshima, Japan) and subsequently frozen via freeze-spraying. The frozen tissue block was sectioned into 10 μm slices using a cryostat (CryoStar™ NX70, Epredia, Portsmouth NH, USA), with the chamber temperature maintained at − 25 °C and the object holder at − 23 °C. The sections were carefully placed on slides coated with indium tin oxide (ITO, Bruker Daltonics, Billerica, MA, USA) for subsequent MSI analysis. Prior to Nano-PALDI MSI analysis, optical images of the tissues were captured using a microscope scanner (Nanozoomer, Hamamatsu Photonics, Shizuoka, Japan). For MSI analysis, DTTC, NPGDC, and TMPIS were evaluated.

### MSI by nano-PALDI method

Target MS plates were coated with a 10 mg mL^− 1^ solution of matrix nanoparticles (NPs) for Nano-PALDI. NPs were prepared by adding an aqueous solution of FeCl_2_・4H_2_O to 20 mL of γ-aminopropyltriethoxysilane^28^. NPs were sprayed onto the skin tissue sections on the ITO-coated glass slides using an airbrush (nozzle caliber, 0.2 mm). After spraying with an ionization-assisting reagent, sections were analyzed using a MALDI-qTOF-MS (tims TOF flex, Bruker Daltonics, Billerica, MA, USA) with a 10-kHz pulse laser. The laser spot areas (200 shots) were analyzed with a spot-to-spot center distance of 5 μm in each direction on the section, and spectra were acquired across *m/z* 400–1200. The tissue surface was irradiated with YAG laser shots in the positive ion detection mode, with optimized power to minimize in-source decay of the targets. The obtained MS spectra were reconstructed into MS images with a mass bin width of *m/z* ± 0.1 from the exact mass using SCiLS™ Lab software (Bruker Daltonics, Billerica, MA, USA).

### Preparation and measurement of lipid matrix models

The thermal behavior of lipid matrix models was studied using differential scanning calorimetry (DSC). Lipid matrix models of SC intercellular lipids were prepared by mixing ceramide III, cholesterol, and palmitic acid in a 1:1:1 molar ratio as described previously^29^. For the fluidity evaluation, 10%, 20%, and 40% (w/w) of each sample and an excess amount of water were added to the lipid matrix models, followed by heating to 100 °C for 10 min while being treated with a probe-type ultrasonic homogenizer (UD-100, TOMY SEIKO, Tokyo, Japan). After heating, the samples were cooled to room temperature; heating-cooling cycles were performed twice to ensure thorough mixing and lipid incorporation. DSC measurements (DSC-7000X, Hitachi, Tokyo, Japan) were conducted from − 30 to 120 °C at a heating rate of 10 °C/min to observe any shifts in the phase transition peaks. Oils tested included DTTC, GO, IPM, MO, NPGDC, SQ, TEH, TMPIS, and TO. Log *K*_*ow*_ values were calculated using EPI Suite™ (ver. 4.1, United States Environmental Protection Agency, Washington, DC, USA).

### Statistical analysis

Analyses were performed using JMP^®^ 18 (JMP Statistical Discovery LLC, Cary, NC, USA).

## Conclusion

This study suggested a plausible mechanism underlying the superior moisturizing performance of the novel ester oil, DTTC. Our findings demonstrate that its sustained hydrating effect, observed even after wash-off, arises not from simple surface occlusion but from a dual mechanism: (1) penetration into and retention within the SC and (2) highly compatible integration with the intercellular lipid matrix without substantial structural disruption. This integrative mechanism, in which the emollient acts in concert with the skin’s native architecture, provides a rationale for its ability to enhance water-retaining capacity. It distinguishes DTTC from classic occlusives that act only at the surface and from other emollients that may be less effective with respect to penetration and/or structural compatibility. Collectively, these results position DTTC as a high-performance emollient and provide a new framework for the rational design of advanced skincare ingredients that deliver long-lasting hydration by functioning in harmony with the SC structure.

## Supplementary Information

Below is the link to the electronic supplementary material.

Supplementary Material 1

## Data Availability

The authors confirm that the data supporting the findings of this study are available within the article.
