# Supplementary material for: Emollient ditrimethylolpropane tetracaprylate penetrates the stratum corneum and maintains hydration without disrupting lipid structure
Source: Sci Rep. 2026 Jun 5;16:22405. doi: 10.1038/s41598-026-52849-1 (PMC13376355; doi:10.1038/s41598-026-52849-1)
Supplement: Supplementary file 1 — Supplementary Material 1 [file 41598_2026_52849_MOESM1_ESM.docx]

**Emollient ditrimethylolpropane tetracaprylate penetrates the stratum corneum and maintains hydration without disrupting lipid structure**

Yukiko Suzuki Uemura, Naoko Hanada Yamazaki, Manaka Honda, Toshinori Saida, Keiichi Oyama and Yoshihiro Tokudome^*^

**Supplementary information**

**
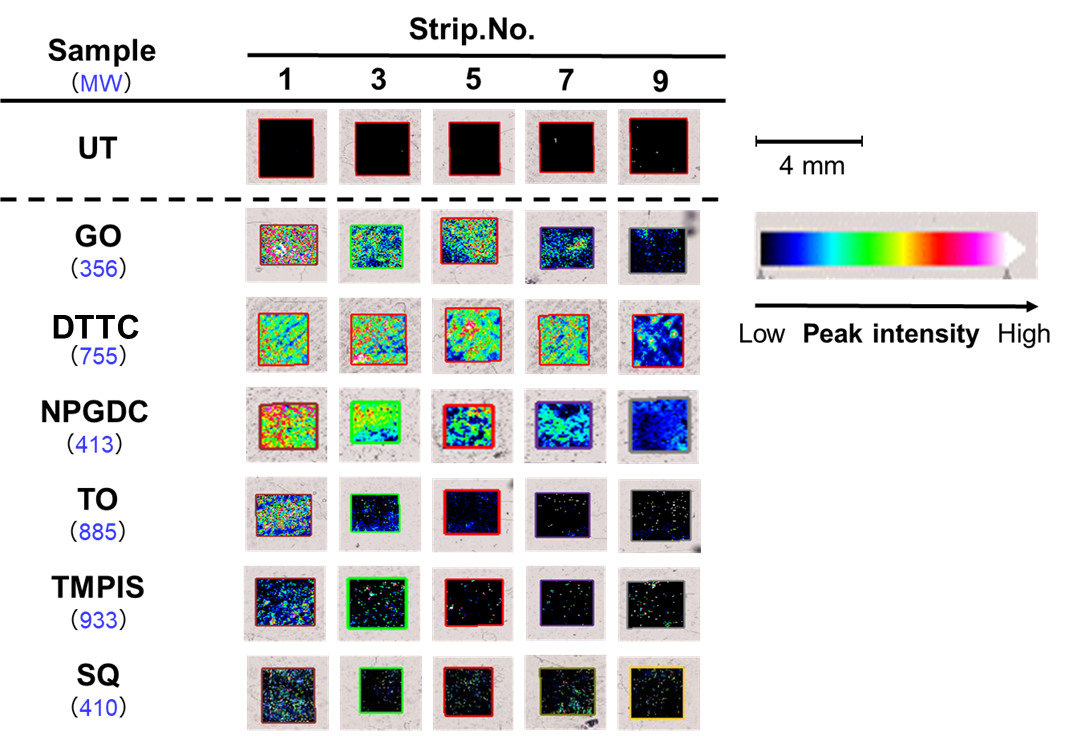
**

**Figure S1.** Nano-PALDI MSI of stratum corneum (SC) after in vivo treatment and tape stripping. Healthy subjects received forearm applications of the test oils for 1 h as described in Methods (*In vivo* skin hydration study). One hour after washing with a non-detergent soap, SC was collected from the treated sites by tape stripping. Untreated skin (UT) served as a negative control. Tape-stripped SC was mounted on ITO-coated glass slides and optical images were acquired prior to MSI to register the SC areas. The o-phthalaldehyde (OPA) assay confirmed comparable total protein content per tape across conditions, indicating similar tape-stripping depth; no statistically significant differences were observed between UT and oil-treated sites or among oil types (data not shown). MSI was performed as described in Methods (MSI by Nano-PALDI method). Signals assigned to the applied oils and/or their characteristic ions (exact masses listed in Methods) are shown as ion-intensity heatmaps overlaid on the optical images. Color bars indicate normalized intensity (0–100%; normalized per image).


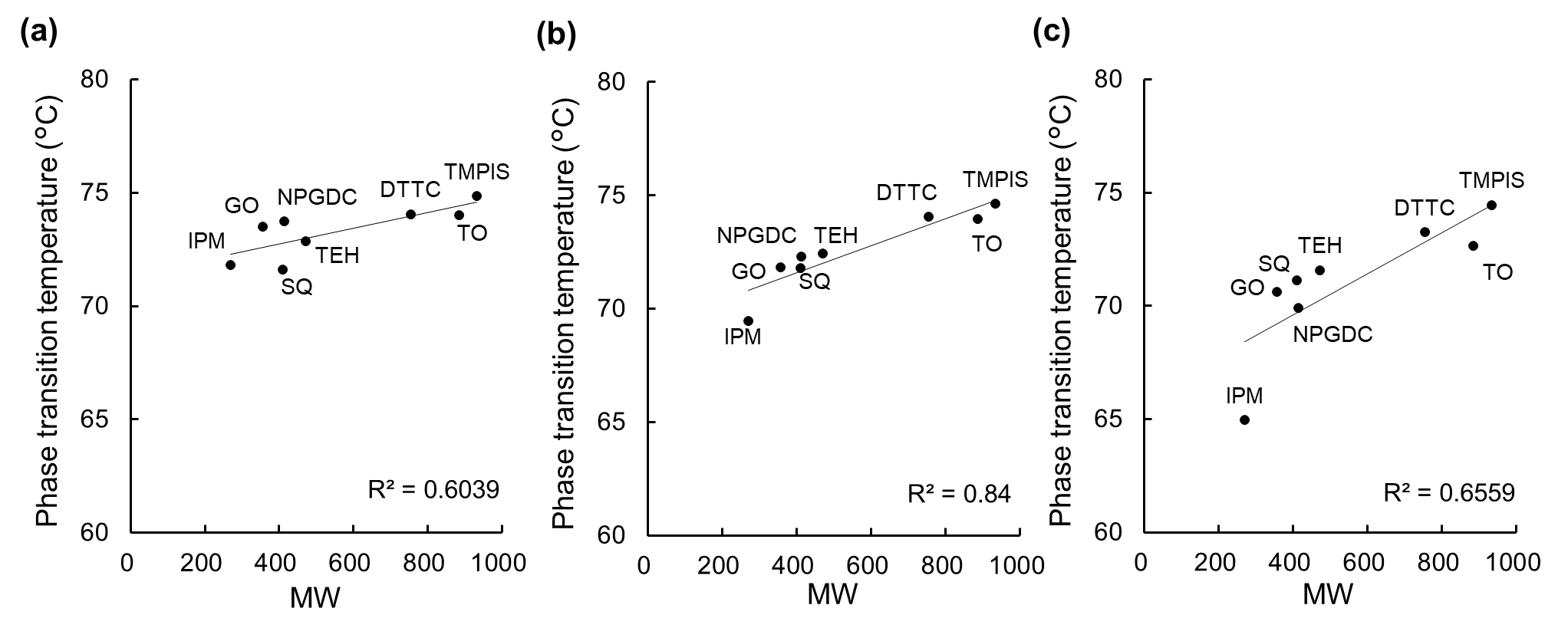


**Figure S2.** Correlation between SC lipid phase transition temperature and MW of systems containing **(a)** 10%, **(b)** 20%, and **(c)** 40% oil agents.


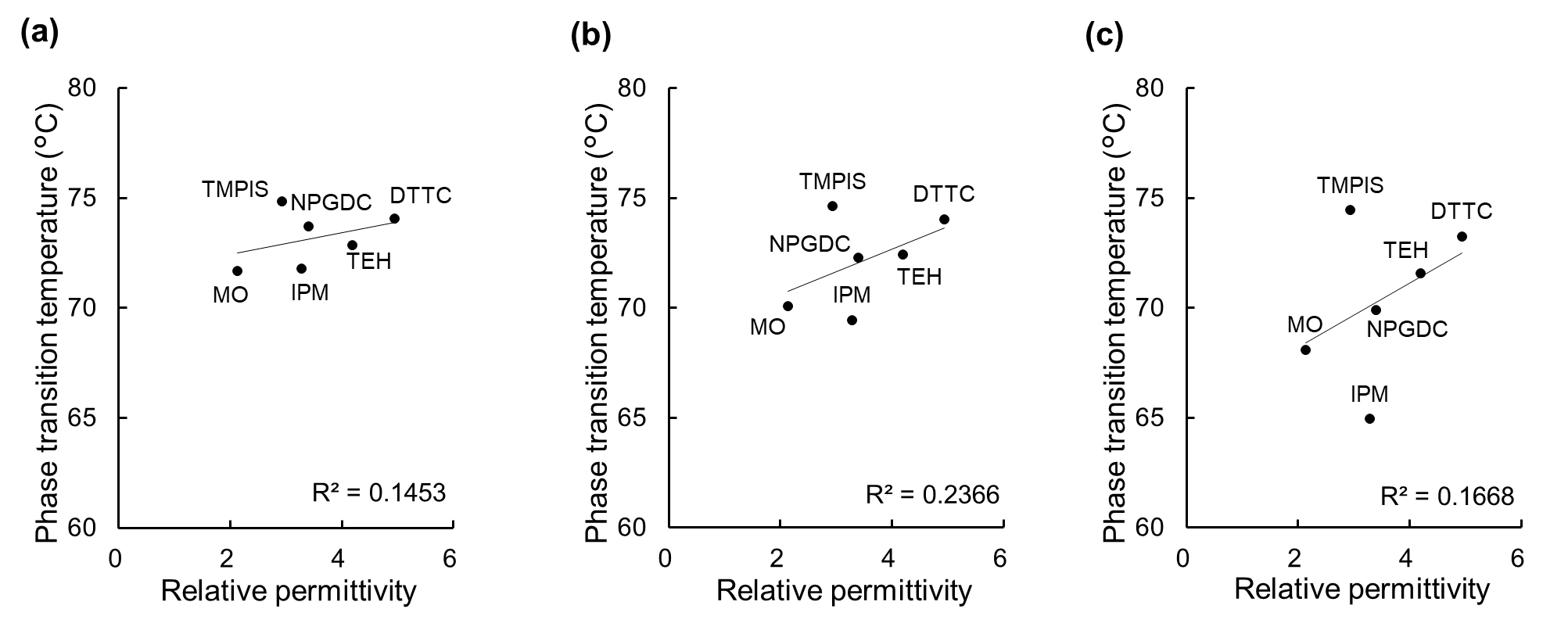


**Figure S3.** Correlation between SC lipid phase transition temperature and oil permittivity of systems containing **(a)** 10%, **(b)** 20%, and **(c)** 40% oil agents.


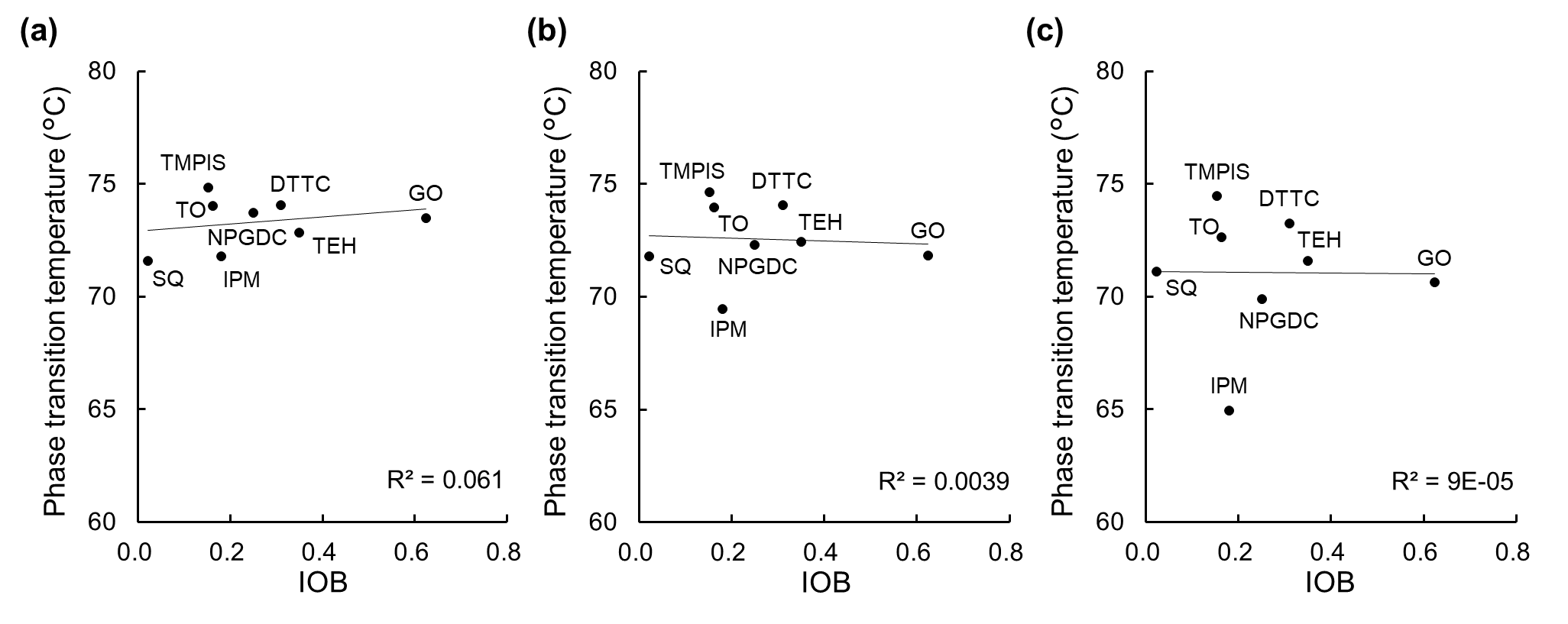


**Figure S4.** Correlation between SC lipid phase transition temperature and inorganic/organic balance values of oil agents in systems containing **(a)** 10%, **(b)** 20%, and **(c)** 40% oil agents.

**
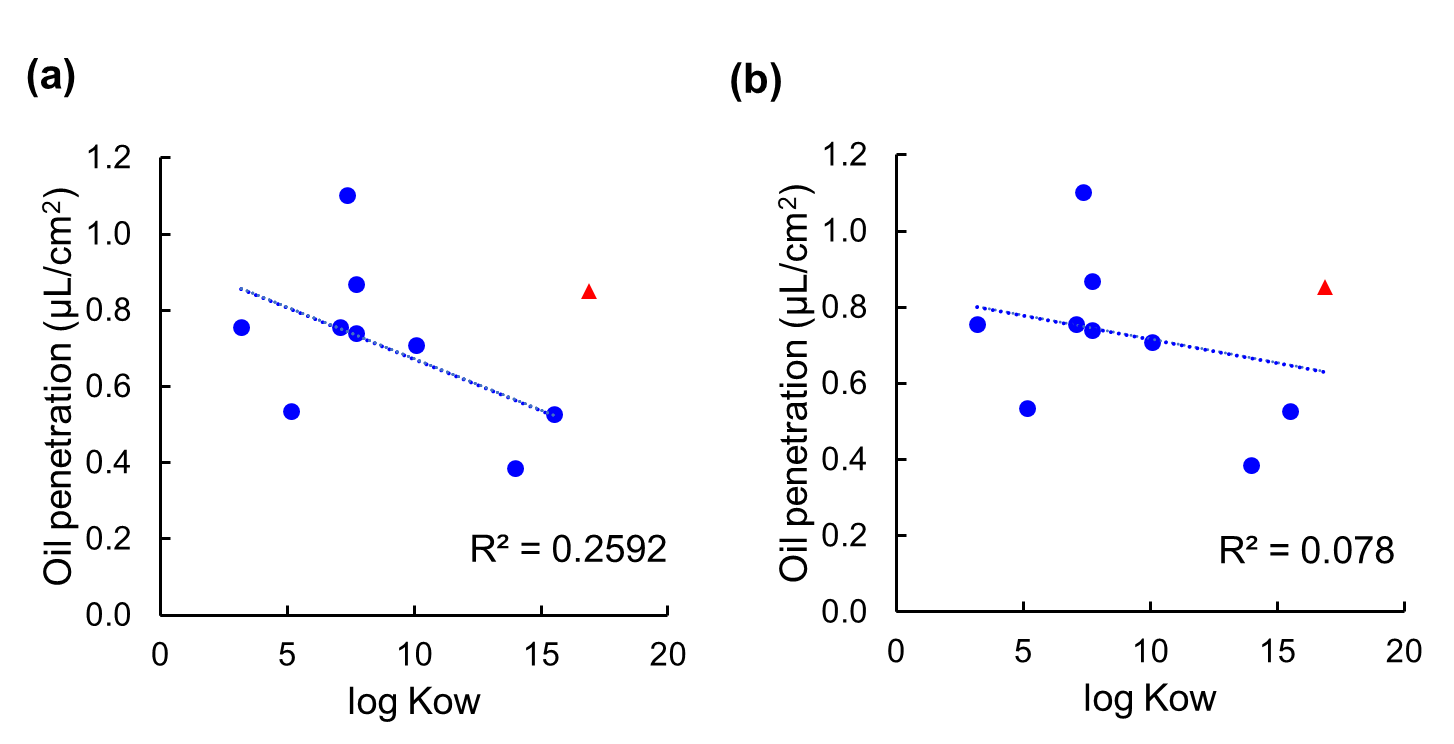
**

**
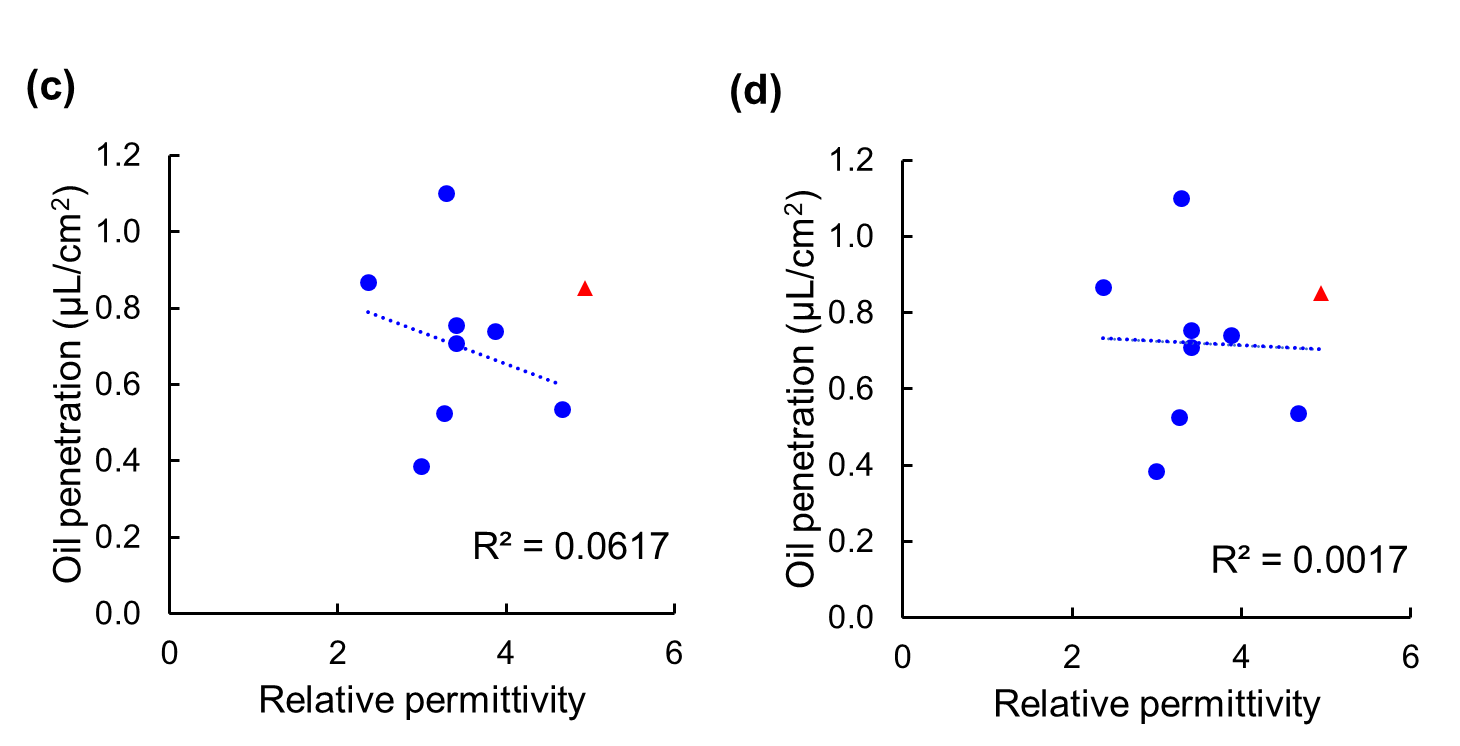
**

**
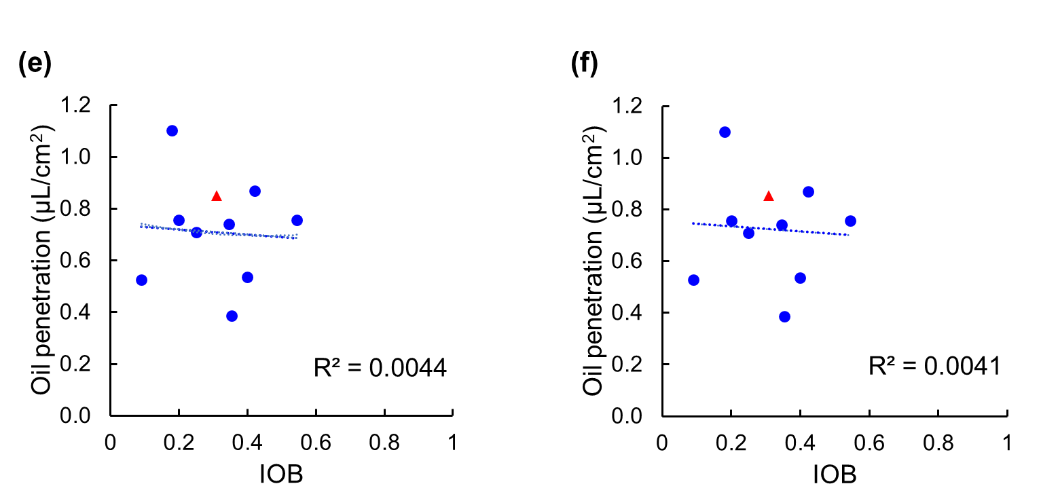
**

**Figure S5.** Relationship between **(a, b)** molecular weight, **(c, d)** relative permittivity, and **(e, f)** IOB and skin penetration at 24 h. Conventional oil agents are plotted as blue circles and DTTC as a red triangle. Regression lines and corresponding R^2^ values are shown for conventional oil agents **(a, c, e)** and all oil agents **(b, d, f)**, respectively.
